# OASIS STUDY: ACTIVITY PATTERNS OF OLDER ADULTS LIVING IN FIVE AGING IN PLACE COMMUNITIES

**DOI:** 10.1093/geroni/igz038.3551

**Published:** 2019-11-08

**Authors:** Vincent DePaul, Catherine Donnelly, Simone Parniak, Oasis Study Collaborative

**Affiliations:** 1 Queen's University, Kingston, Ontario, Canada; 2 Oasis Study Collaboration, Ontario, Canada

## Abstract

Background: The Oasis program is a model of aging-in-place that targets social connectedness, physical activity and nutritional wellness through member-driven programming. Oasis, first established in an apartment building in Kingston Ontario, has recently been expanded to 6 new communities across Ontario in a participatory action research study. The purpose of this poster is to describe the physical activity patterns of five unique Oasis communities (Original and 4 new) and explore the impact of personal, environmental, and Oasis program characteristics on these patterns. Methods: Participants were recruited from Oasis communities in 3 market-priced apartiments, 1 subsidized apartment, and 1 mobile-home park. Participants wore the ActivPAL3 activity monitor for 7 days. Mobility was measured using the Timed Up and Go. Programming was described by type (e.g. social, exercise, nutrition), frequency, and timing of programming. Results: Participants included 70 older adults aged 79.8(min 62, max 97), community mean age ranged from 66.2 – 83.5 years. TUG score 11.6(SD 4.9) (community range 10.5 to 13.7 s). Average daily step count was 5800(SD 2835) steps, with communities ranging from 4685 to 6472 steps/day. An average of 604(community range 236 – 1056) of steps were taken at a healthy pace(100 steps/min). Only 27% of participants took the recommended 7000 steps/day (with community rates ranging from 9.5% to 37.5%). Conclusions: Older adults within these aging in place communities demonstrated low to moderate levels of physical activity, with activity patterns differing across communities. Impact of community make up and characteristics on activity patterns will be presented.

